# Crystal structure of di-μ-benzato-κ^4^ *O*:*O*′-bis­[aqua­(benzato-κ*O*)(benzato-κ^2^ *O*,*O*′)(2,2′:6′,2′′-terpyridine-κ^3^ *N*,*N*′,*N*′′)europium(III)]–benzoic acid (1/2)

**DOI:** 10.1107/S1600536814018182

**Published:** 2014-08-13

**Authors:** Frankie White, Richard E. Sykora

**Affiliations:** a University of South Alabama, Department of Chemistry, Mobile, AL 36688-0002, USA

**Keywords:** crystal structure, dinuclear europium(III) complex, 2,2′:6′,2′′-terpyridine, benzoate, hydrogen bonding

## Abstract

The title compound, [Eu_2_(C_7_H_5_O_2_)_6_(C_15_H_11_N_3_)_2_(H_2_O)_2_]·2C_7_H_6_O_2_, is a co-crystalline compound containing a dinuclear Eu^III^ coordination complex with inversion symmetry co-crystallized with benzoic acid in a 1:2 ratio. The Eu^3+^ ions within the dimer are nine-coordinate, containing one tridentate terpyridine, one water, and four benzoate ions, two of which bridge the Eu^3+^ ions. Of the four benzoate ligands coordinating to each Eu^3+^ position, three distinct coordination modes [monodentate, bidentate–chelating, and bidentate–bridging (twice)] are observed. Within the crystal, there are two additional uncoordinating benzoic acid mol­ecules per dinuclear complex. Within the dimer, the water bound to each Eu^3+^ ion participates in intra­molecular hydrogen bonding with a coordinating benzoate. Additionally, the carb­oxy­lic acid group on the benzoic acid participates in inter­molecular hydrogen bonding with a benzoate ligand bound to the dimer complex.

## Related literature   

Coordination of lanthanide ions by organic ligands has many uses including solvent extractions from nuclear waste and light emitting diodes in electronics (Dul *et al.*, 2013 ▶; Romero *et al.*, 2012 ▶). Organic ligands are also capable of increasing the intensity of lanthanide emissions (Romero *et al.*, 2012 ▶). The title compound, and similar derivatives, are of inter­est because of the effect these organic ligands can have on increasing the emission intensity from lanthanide ions in white-light-emitting phosphors. For lanthanide–terpyridine complex chemistry, see: Frost *et al.* (1969 ▶). For synthesis, structural, and spectroscopic properties of related lanthanide complexes containing both terpyridine and bridging benzoate ligands, see: Messimeri *et al.* (2007 ▶); Fiedler *et al.* (2007 ▶).
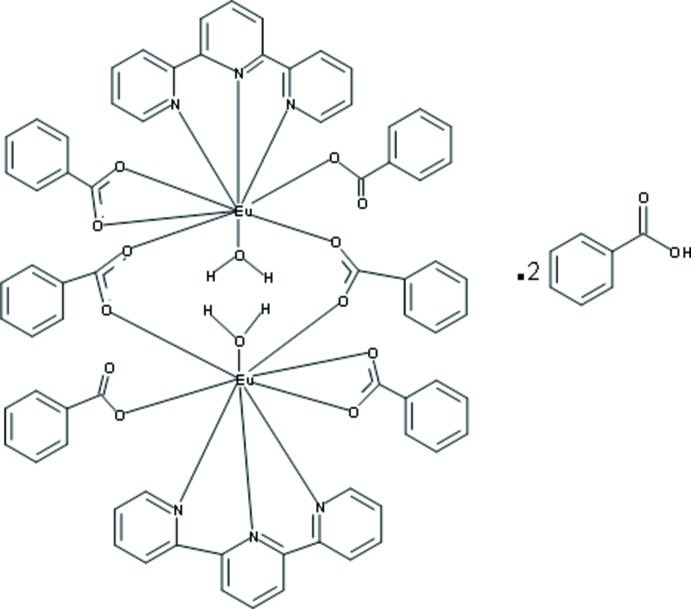


## Experimental   

### Crystal data  

[Eu_2_(C_7_H_5_O_2_)_6_(C_15_H_11_N_3_)_2_(H_2_O)_2_]·2C_7_H_6_O_2_
*M* *_r_* = 1777.38
Triclinic, 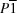

*a* = 11.8435 (5) Å
*b* = 13.9470 (7) Å
*c* = 14.0090 (7) Å
α = 102.568 (4)°
β = 111.400 (5)°
γ = 108.583 (5)°
*V* = 1890.40 (16) Å^3^
*Z* = 1
Mo *K*α radiation
μ = 1.72 mm^−1^
*T* = 180 K
0.05 × 0.04 × 0.03 mm

### Data collection  

Agilent Xcalibur Eos diffractometer
Absorption correction: multi-scan (*CrysAlis PRO*; Agilent, 2014 ▶) *T*_min_ = 0.988, *T*_max_ = 1.000
12682 measured reflections
6921 independent reflections
5919 reflections with *I* > 2σ(*I*)
*R*_int_ = 0.037

### Refinement  

*R*[*F* ^2^ > 2σ(*F* ^2^)] = 0.039
*wR*(*F* ^2^) = 0.070
*S* = 1.03
6921 reflections
507 parameters
H-atom parameters constrained
Δρ_max_ = 0.63 e Å^−3^
Δρ_min_ = −0.53 e Å^−3^

### 

Data collection: *CrysAlis PRO* (Agilent, 2014 ▶); cell refinement: *CrysAlis PRO*; data reduction: *CrysAlis PRO*; program(s) used to solve structure: *SHELXS97* (Sheldrick, 2008 ▶); program(s) used to refine structure: *SHELXL97* (Sheldrick, 2008 ▶); molecular graphics: *OLEX2* (Dolomanov *et al.*, 2009 ▶); software used to prepare material for publication: *OLEX2* and *publCIF* (Westrip, 2010 ▶).

## Supplementary Material

Crystal structure: contains datablock(s) I, New_Global_Publ_Block. DOI: 10.1107/S1600536814018182/pj2014sup1.cif

Structure factors: contains datablock(s) I. DOI: 10.1107/S1600536814018182/pj2014Isup2.hkl

**Fig. 1.**
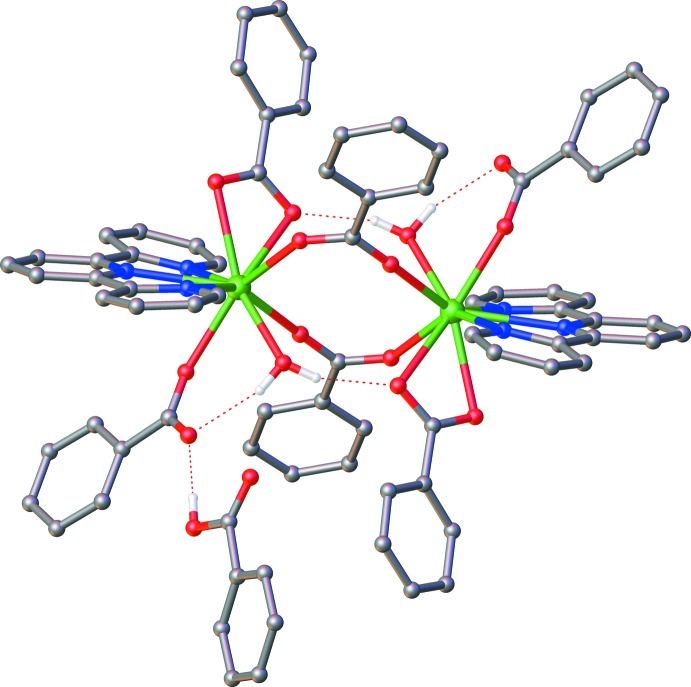
A ball-and-stick representaion of the molecular structure of I. Hydrogen atoms on the aromatic rings have been removed for clarity.

I . DOI: 10.1107/S1600536814018182/pj2014fig1.tif
A ball-and-stick representaion of the mol­ecular structure of **I**. Hydrogen atoms on the aromatic rings have been removed for clarity.

CCDC reference: 1018455

Additional supporting information:  crystallographic information; 3D view; checkCIF report

## Figures and Tables

**Table 1:** Hydrogen-bond geometry (Å, °)

*D*—H⋯*A*	*D*—H	H⋯*A*	*D*⋯*A*	*D*—H⋯*A*
O6—H6⋯O8^i^	0.82	1.76	2.582 (4)	178
O5—H5*A*⋯O8	0.87	1.98	2.796 (4)	155
O5—H5*B*⋯O4^ii^	0.87	1.95	2.768 (4)	155

Symmetry codes: (i) 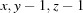; (ii) 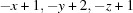.

## supplementary crystallographic information

### S1. Synthesis and crystallization

Methanol solutions consisting of 0.1 *M* EuCl_3_ (1mL), 0.1 *M* benzoic acid (1 mL), and 0.1 *M* 2,2':6',2"-terpyridine (1 mL) were mixed in a test tube. The resultant solution was left to evaporate under normal atmospheric conditions which resulted in the formation of colorless crystals. The single crystals were gathered and isolated for studies.

### S2. Refinement

Crystal data, data collection and structure refinement details are summarized in Table 1. H-atoms were placed in calculated positions using the hadd command in *Olex2* (Dolomanov *et al.*, 2009) and allowed to ride during subsequent refinement, with *U*_iso_(H) = 1.2*U*_eq_(C) and C—H distances of 0.93 Å for ring hydrogens, with *U*_iso_(H) = 1.5*U*_eq_(O) and O—H distances of 0.875 Å for water hydrogens, and with *U*_iso_(H) = 1.5*U*_eq_(O) and an O—H distance of 0.82 Å for the carb­oxy­lic acid hydrogen. Free refinement of O—H hydrogen atoms resulted in reasonable geometries, but unexpectedly short O—H bond distances.

### Crystal data

**Table:**

[Eu_2_(C_7_H_5_O_2_)_6_(C_15_H_11_N_3_)_2_(H_2_O)_2_]·2C_7_H_6_O_2_	*Z* = 1
*M**_r_* = 1777.38	*F*(000) = 896
Triclinic, *P*1	*D*_x_ = 1.561 Mg m^−^^3^
*a* = 11.8435 (5) Å	Mo *K*α radiation, λ = 0.71073 Å
*b* = 13.9470 (7) Å	Cell parameters from 5349 reflections
*c* = 14.0090 (7) Å	θ = 3.3–26.4°
α = 102.568 (4)°	µ = 1.72 mm^−^^1^
β = 111.400 (5)°	*T* = 180 K
γ = 108.583 (5)°	Prism, clear colourless
*V* = 1890.40 (16) Å^3^	0.05 × 0.04 × 0.03 mm

### Data collection

**Table:**

Agilent Xcalibur Eos diffractometer	6921 independent reflections
Radiation source: Enhance (Mo) X-ray Source	5919 reflections with *I* > 2σ(*I*)
Graphite monochromator	*R*_int_ = 0.037
Detector resolution: 16.0514 pixels mm^-1^	θ_max_ = 25.4°, θ_min_ = 3.0°
ω scans	*h* = −14→14
Absorption correction: multi-scan (*CrysAlis PRO*; Agilent, 2014)	*k* = −16→16
*T*_min_ = 0.988, *T*_max_ = 1.000	*l* = −16→15
12682 measured reflections	

### Refinement

**Table:**

Refinement on *F*^2^	Primary atom site location: heavy-atom method
Least-squares matrix: full	Secondary atom site location: difference Fourier map
*R*[*F*^2^ > 2σ(*F*^2^)] = 0.039	Hydrogen site location: inferred from neighbouring sites
*wR*(*F*^2^) = 0.070	H-atom parameters constrained
*S* = 1.03	*w* = 1/[σ^2^(*F*_o_^2^) + (0.0205*P*)^2^] where *P* = (*F*_o_^2^ + 2*F*_c_^2^)/3
6921 reflections	(Δ/σ)_max_ = 0.001
507 parameters	Δρ_max_ = 0.63 e Å^−^^3^
0 restraints	Δρ_min_ = −0.53 e Å^−^^3^

### Special details

**Table:**

Geometry. All e.s.d.'s (except the e.s.d. in the dihedral angle between two l.s. planes) are estimated using the full covariance matrix. The cell e.s.d.'s are taken into account individually in the estimation of e.s.d.'s in distances, angles and torsion angles; correlations between e.s.d.'s in cell parameters are only used when they are defined by crystal symmetry. An approximate (isotropic) treatment of cell e.s.d.'s is used for estimating e.s.d.'s involving l.s. planes.
Refinement. Refinement of *F*^2^ against ALL reflections. The weighted *R*-factor *wR* and goodness of fit *S* are based on *F*^2^, conventional *R*-factors *R* are based on *F*, with *F* set to zero for negative *F*^2^. The threshold expression of *F*^2^ > 2σ(*F*^2^) is used only for calculating *R*-factors(gt) *etc*. and is not relevant to the choice of reflections for refinement. *R*-factors based on *F*^2^ are statistically about twice as large as those based on *F*, and *R*-factors based on ALL data will be even larger.

### Fractional atomic coordinates and isotropic or equivalent isotropic displacement parameters (Å^2^)

**Table:**

	*x*	*y*	*z*	*U*_iso_*/*U*_eq_	
Eu1	0.60725 (2)	0.901818 (18)	0.607785 (17)	0.01686 (7)	
N3	0.4478 (3)	0.7011 (3)	0.5632 (3)	0.0206 (8)	
O3	0.6498 (3)	0.7737 (2)	0.4829 (2)	0.0231 (7)	
O9	0.5811 (3)	0.9085 (2)	0.7688 (2)	0.0237 (7)	
O4	0.4730 (3)	0.8045 (2)	0.4030 (2)	0.0231 (7)	
O1	0.7127 (3)	1.0054 (2)	0.5254 (2)	0.0289 (7)	
O8	0.7071 (3)	1.0699 (3)	0.9073 (2)	0.0356 (8)	
N1	0.8631 (3)	0.9976 (3)	0.7433 (3)	0.0241 (8)	
O7	0.8924 (4)	0.3302 (3)	−0.0132 (3)	0.0461 (9)	
N2	0.7144 (3)	0.7916 (3)	0.7133 (2)	0.0186 (8)	
C6	0.8428 (4)	0.8412 (4)	0.7946 (3)	0.0218 (10)	
C25	0.3769 (4)	0.6313 (3)	0.2020 (3)	0.0246 (10)	
H25	0.3164	0.6570	0.2107	0.030*	
C17	0.7978 (4)	1.1095 (3)	0.4350 (3)	0.0211 (10)	
C24	0.5068 (4)	0.6743 (3)	0.2884 (3)	0.0205 (9)	
C23	0.5477 (4)	0.7567 (3)	0.3978 (3)	0.0187 (9)	
C16	0.6934 (4)	1.0641 (3)	0.4701 (3)	0.0196 (9)	
C10	0.6377 (4)	0.6881 (4)	0.6939 (3)	0.0212 (10)	
C1	0.9367 (4)	1.0985 (4)	0.7559 (4)	0.0330 (11)	
H1	0.8960	1.1277	0.7075	0.040*	
C31	0.9065 (4)	0.4374 (4)	0.1515 (3)	0.0254 (10)	
C38	0.5944 (4)	0.9156 (3)	0.9417 (3)	0.0202 (9)	
C43	0.5257 (4)	0.8029 (4)	0.9017 (3)	0.0255 (10)	
H43	0.4975	0.7619	0.8285	0.031*	
C37	0.6298 (4)	0.9697 (4)	0.8685 (3)	0.0237 (10)	
C29	0.5979 (4)	0.6380 (4)	0.2737 (3)	0.0267 (11)	
H29	0.6857	0.6673	0.3305	0.032*	
C3	1.1281 (5)	1.1201 (4)	0.9069 (4)	0.0408 (13)	
H3	1.2166	1.1616	0.9628	0.049*	
C42	0.4985 (4)	0.7505 (4)	0.9696 (4)	0.0330 (11)	
H42	0.4526	0.6747	0.9420	0.040*	
C15	0.3195 (4)	0.6563 (4)	0.4855 (4)	0.0293 (11)	
H15	0.2864	0.7014	0.4568	0.035*	
C11	0.4957 (4)	0.6357 (3)	0.6054 (3)	0.0219 (10)	
C39	0.6353 (5)	0.9755 (4)	1.0512 (3)	0.0304 (11)	
H39	0.6821	1.0512	1.0795	0.036*	
C41	0.5395 (5)	0.8111 (4)	1.0781 (4)	0.0386 (13)	
H41	0.5217	0.7761	1.1239	0.046*	
C26	0.3368 (5)	0.5508 (4)	0.1032 (3)	0.0300 (11)	
H26	0.2493	0.5213	0.0459	0.036*	
C40	0.6065 (5)	0.9228 (4)	1.1184 (4)	0.0402 (13)	
H40	0.6329	0.9634	1.1913	0.048*	
C4	1.0557 (5)	1.0153 (4)	0.8952 (4)	0.0350 (12)	
H4	1.0955	0.9850	0.9427	0.042*	
C13	0.2820 (5)	0.4796 (4)	0.4873 (4)	0.0411 (13)	
H13	0.2268	0.4054	0.4622	0.049*	
C2	1.0678 (5)	1.1624 (4)	0.8350 (4)	0.0378 (12)	
H2	1.1147	1.2324	0.8398	0.045*	
C7	0.8972 (5)	0.7876 (4)	0.8584 (4)	0.0298 (11)	
H7	0.9861	0.8232	0.9145	0.036*	
C5	0.9242 (4)	0.9556 (4)	0.8130 (3)	0.0213 (10)	
C14	0.2330 (5)	0.5469 (4)	0.4451 (4)	0.0330 (11)	
H14	0.1441	0.5192	0.3908	0.040*	
C12	0.4144 (5)	0.5249 (4)	0.5675 (4)	0.0320 (11)	
H12	0.4495	0.4808	0.5965	0.038*	
C28	0.5573 (5)	0.5576 (4)	0.1736 (4)	0.0376 (13)	
H28	0.6180	0.5332	0.1632	0.045*	
C32	0.8532 (5)	0.4409 (4)	0.2239 (4)	0.0373 (12)	
H32	0.7912	0.3768	0.2192	0.045*	
C18	0.9132 (5)	1.0938 (4)	0.4702 (4)	0.0382 (13)	
H18	0.9255	1.0536	0.5148	0.046*	
C9	0.6876 (5)	0.6307 (4)	0.7557 (4)	0.0314 (11)	
H9	0.6329	0.5590	0.7414	0.038*	
C36	0.9973 (5)	0.5325 (4)	0.1575 (4)	0.0413 (13)	
H36	1.0320	0.5300	0.1077	0.050*	
C8	0.8181 (5)	0.6823 (4)	0.8371 (4)	0.0351 (12)	
H8	0.8533	0.6454	0.8784	0.042*	
C27	0.4275 (5)	0.5144 (4)	0.0903 (4)	0.0370 (12)	
H27	0.4004	0.4597	0.0240	0.044*	
C35	1.0371 (6)	0.6320 (5)	0.2375 (5)	0.0530 (15)	
H35	1.0995	0.6961	0.2425	0.064*	
C22	0.7788 (5)	1.1673 (4)	0.3664 (4)	0.0374 (12)	
H22	0.7011	1.1780	0.3416	0.045*	
C30	0.8641 (4)	0.3329 (4)	0.0611 (4)	0.0272 (11)	
C33	0.8922 (5)	0.5406 (5)	0.3042 (4)	0.0482 (15)	
H33	0.8570	0.5434	0.3536	0.058*	
C21	0.8756 (7)	1.2088 (4)	0.3351 (5)	0.0632 (19)	
H21	0.8627	1.2476	0.2891	0.076*	
C19	1.0107 (5)	1.1375 (5)	0.4396 (5)	0.0595 (18)	
H19	1.0897	1.1288	0.4654	0.071*	
C20	0.9896 (7)	1.1939 (5)	0.3702 (6)	0.072 (2)	
H20	1.0534	1.2218	0.3476	0.087*	
C34	0.9832 (6)	0.6347 (5)	0.3095 (5)	0.0561 (17)	
H34	1.0088	0.7014	0.3626	0.067*	
O6	0.7974 (3)	0.2460 (2)	0.0747 (2)	0.0333 (8)	
H6	0.7697	0.1911	0.0207	0.050*	
O5	0.6709 (3)	1.0977 (2)	0.7081 (2)	0.0263 (7)	
H5A	0.6854	1.1097	0.7764	0.040*	
H5B	0.6061	1.1148	0.6754	0.040*	
O2	0.5972 (3)	1.0882 (2)	0.4455 (2)	0.0302 (7)	

### Atomic displacement parameters (Å^2^)

**Table:**

	*U*^11^	*U*^22^	*U*^33^	*U*^12^	*U*^13^	*U*^23^
Eu1	0.01700 (12)	0.01815 (12)	0.01788 (12)	0.00895 (9)	0.00798 (10)	0.00955 (9)
N3	0.021 (2)	0.019 (2)	0.0224 (19)	0.0092 (18)	0.0100 (18)	0.0103 (16)
O3	0.0210 (17)	0.0307 (19)	0.0190 (15)	0.0155 (15)	0.0060 (14)	0.0113 (14)
O9	0.0325 (19)	0.0246 (18)	0.0174 (15)	0.0147 (16)	0.0128 (15)	0.0088 (14)
O4	0.0252 (17)	0.0274 (18)	0.0242 (16)	0.0180 (15)	0.0124 (15)	0.0120 (14)
O1	0.0322 (19)	0.032 (2)	0.0327 (18)	0.0157 (17)	0.0195 (16)	0.0200 (16)
O8	0.050 (2)	0.021 (2)	0.0271 (18)	0.0048 (18)	0.0208 (18)	0.0072 (15)
N1	0.019 (2)	0.021 (2)	0.028 (2)	0.0067 (18)	0.0062 (18)	0.0122 (17)
O7	0.063 (3)	0.040 (2)	0.040 (2)	0.016 (2)	0.032 (2)	0.0177 (18)
N2	0.020 (2)	0.022 (2)	0.0185 (19)	0.0127 (18)	0.0107 (18)	0.0089 (16)
C6	0.024 (3)	0.028 (3)	0.021 (2)	0.016 (2)	0.012 (2)	0.013 (2)
C25	0.032 (3)	0.024 (3)	0.028 (2)	0.015 (2)	0.017 (2)	0.017 (2)
C17	0.028 (3)	0.019 (2)	0.022 (2)	0.010 (2)	0.017 (2)	0.0066 (19)
C24	0.026 (3)	0.019 (2)	0.021 (2)	0.010 (2)	0.012 (2)	0.013 (2)
C23	0.023 (2)	0.018 (2)	0.020 (2)	0.007 (2)	0.013 (2)	0.0112 (19)
C16	0.018 (2)	0.017 (2)	0.016 (2)	0.006 (2)	0.005 (2)	0.0009 (19)
C10	0.025 (3)	0.024 (3)	0.024 (2)	0.014 (2)	0.016 (2)	0.012 (2)
C1	0.023 (3)	0.027 (3)	0.044 (3)	0.011 (2)	0.007 (2)	0.021 (2)
C31	0.023 (3)	0.024 (3)	0.022 (2)	0.011 (2)	0.002 (2)	0.010 (2)
C38	0.023 (3)	0.023 (3)	0.020 (2)	0.013 (2)	0.011 (2)	0.011 (2)
C43	0.024 (3)	0.029 (3)	0.020 (2)	0.012 (2)	0.008 (2)	0.007 (2)
C37	0.027 (3)	0.024 (3)	0.027 (3)	0.017 (2)	0.014 (2)	0.012 (2)
C29	0.029 (3)	0.033 (3)	0.021 (2)	0.016 (2)	0.014 (2)	0.011 (2)
C3	0.018 (3)	0.038 (3)	0.042 (3)	0.004 (3)	0.000 (2)	0.011 (3)
C42	0.027 (3)	0.028 (3)	0.037 (3)	0.006 (2)	0.010 (2)	0.016 (2)
C15	0.027 (3)	0.029 (3)	0.033 (3)	0.013 (2)	0.012 (2)	0.014 (2)
C11	0.028 (3)	0.022 (3)	0.022 (2)	0.012 (2)	0.016 (2)	0.011 (2)
C39	0.043 (3)	0.021 (3)	0.026 (3)	0.012 (2)	0.017 (2)	0.008 (2)
C41	0.047 (3)	0.046 (4)	0.029 (3)	0.018 (3)	0.022 (3)	0.023 (3)
C26	0.032 (3)	0.024 (3)	0.021 (2)	0.006 (2)	0.005 (2)	0.008 (2)
C40	0.058 (4)	0.039 (4)	0.022 (3)	0.017 (3)	0.020 (3)	0.013 (2)
C4	0.024 (3)	0.038 (3)	0.037 (3)	0.011 (3)	0.005 (2)	0.023 (3)
C13	0.043 (3)	0.026 (3)	0.045 (3)	0.004 (3)	0.018 (3)	0.017 (3)
C2	0.027 (3)	0.033 (3)	0.045 (3)	0.011 (3)	0.007 (3)	0.020 (3)
C7	0.025 (3)	0.031 (3)	0.034 (3)	0.013 (2)	0.011 (2)	0.018 (2)
C5	0.020 (2)	0.027 (3)	0.022 (2)	0.014 (2)	0.012 (2)	0.012 (2)
C14	0.023 (3)	0.029 (3)	0.034 (3)	0.005 (2)	0.007 (2)	0.010 (2)
C12	0.033 (3)	0.023 (3)	0.034 (3)	0.010 (2)	0.010 (3)	0.013 (2)
C28	0.055 (4)	0.047 (3)	0.035 (3)	0.035 (3)	0.031 (3)	0.019 (3)
C32	0.030 (3)	0.035 (3)	0.033 (3)	0.015 (3)	0.005 (3)	0.006 (2)
C18	0.030 (3)	0.038 (3)	0.054 (3)	0.017 (3)	0.026 (3)	0.015 (3)
C9	0.034 (3)	0.025 (3)	0.039 (3)	0.014 (2)	0.016 (3)	0.018 (2)
C36	0.040 (3)	0.034 (3)	0.042 (3)	0.015 (3)	0.010 (3)	0.019 (3)
C8	0.035 (3)	0.038 (3)	0.039 (3)	0.023 (3)	0.012 (3)	0.025 (3)
C27	0.053 (4)	0.036 (3)	0.022 (3)	0.021 (3)	0.019 (3)	0.008 (2)
C35	0.046 (4)	0.032 (4)	0.061 (4)	0.017 (3)	0.005 (3)	0.019 (3)
C22	0.056 (4)	0.026 (3)	0.037 (3)	0.016 (3)	0.030 (3)	0.013 (2)
C30	0.026 (3)	0.022 (3)	0.030 (3)	0.012 (2)	0.007 (2)	0.011 (2)
C33	0.042 (4)	0.049 (4)	0.038 (3)	0.029 (3)	0.005 (3)	0.000 (3)
C21	0.119 (6)	0.035 (4)	0.060 (4)	0.024 (4)	0.071 (5)	0.025 (3)
C19	0.037 (4)	0.056 (4)	0.077 (4)	0.008 (3)	0.042 (4)	0.001 (4)
C20	0.093 (5)	0.032 (4)	0.095 (5)	−0.004 (4)	0.086 (5)	0.003 (3)
C34	0.046 (4)	0.030 (4)	0.053 (4)	0.022 (3)	−0.007 (3)	−0.007 (3)
O6	0.041 (2)	0.0217 (19)	0.0297 (18)	0.0075 (17)	0.0176 (18)	0.0054 (15)
O5	0.0337 (19)	0.0275 (19)	0.0233 (16)	0.0183 (16)	0.0118 (16)	0.0143 (14)
O2	0.0243 (18)	0.036 (2)	0.0354 (18)	0.0183 (17)	0.0139 (16)	0.0135 (16)

### Geometric parameters (Å, º)

**Table:**

Eu1—N3	2.608 (3)	C42—C41	1.378 (6)
Eu1—O3	2.523 (2)	C15—H15	0.9300
Eu1—O9	2.372 (2)	C15—C14	1.379 (6)
Eu1—O4	2.491 (3)	C11—C12	1.386 (6)
Eu1—O1	2.368 (3)	C39—H39	0.9300
Eu1—N1	2.587 (3)	C39—C40	1.385 (6)
Eu1—N2	2.650 (3)	C41—H41	0.9300
Eu1—C23	2.869 (4)	C41—C40	1.371 (7)
Eu1—O5	2.500 (3)	C26—H26	0.9300
Eu1—O2^i^	2.320 (3)	C26—C27	1.376 (6)
N3—C15	1.333 (5)	C40—H40	0.9300
N3—C11	1.349 (5)	C4—H4	0.9300
O3—C23	1.258 (4)	C4—C5	1.376 (6)
O9—C37	1.262 (5)	C13—H13	0.9300
O4—C23	1.278 (4)	C13—C14	1.381 (6)
O1—C16	1.260 (5)	C13—C12	1.376 (6)
O8—C37	1.256 (5)	C2—H2	0.9300
N1—C1	1.330 (5)	C7—H7	0.9300
N1—C5	1.350 (5)	C7—C8	1.360 (6)
O7—C30	1.201 (5)	C14—H14	0.9300
N2—C6	1.346 (5)	C12—H12	0.9300
N2—C10	1.340 (5)	C28—H28	0.9300
C6—C7	1.397 (5)	C28—C27	1.373 (6)
C6—C5	1.484 (6)	C32—H32	0.9300
C25—H25	0.9300	C32—C33	1.394 (6)
C25—C24	1.389 (5)	C18—H18	0.9300
C25—C26	1.381 (5)	C18—C19	1.384 (7)
C17—C16	1.500 (5)	C9—H9	0.9300
C17—C18	1.380 (5)	C9—C8	1.361 (6)
C17—C22	1.385 (6)	C36—H36	0.9300
C24—C23	1.497 (5)	C36—C35	1.387 (7)
C24—C29	1.390 (5)	C8—H8	0.9300
C16—O2	1.241 (4)	C27—H27	0.9300
C10—C11	1.484 (6)	C35—H35	0.9300
C10—C9	1.405 (5)	C35—C34	1.376 (7)
C1—H1	0.9300	C22—H22	0.9300
C1—C2	1.366 (6)	C22—C21	1.377 (7)
C31—C32	1.376 (6)	C30—O6	1.316 (5)
C31—C36	1.378 (6)	C33—H33	0.9300
C31—C30	1.499 (6)	C33—C34	1.373 (8)
C38—C43	1.384 (6)	C21—H21	0.9300
C38—C37	1.498 (5)	C21—C20	1.358 (8)
C38—C39	1.389 (5)	C19—H19	0.9300
C43—H43	0.9300	C19—C20	1.381 (8)
C43—C42	1.384 (6)	C20—H20	0.9300
C29—H29	0.9300	C34—H34	0.9300
C29—C28	1.390 (6)	O6—H6	0.8200
C3—H3	0.9300	O5—H5A	0.8741
C3—C4	1.375 (6)	O5—H5B	0.8737
C3—C2	1.369 (6)	O2—Eu1^i^	2.319 (3)
C42—H42	0.9300		
			
N3—Eu1—N2	62.51 (10)	C24—C29—C28	119.7 (4)
N3—Eu1—C23	68.56 (10)	C28—C29—H29	120.2
O3—Eu1—N3	71.20 (9)	C4—C3—H3	120.5
O3—Eu1—N1	88.58 (9)	C2—C3—H3	120.5
O3—Eu1—N2	69.88 (8)	C2—C3—C4	119.0 (5)
O3—Eu1—C23	25.99 (9)	C43—C42—H42	120.1
O9—Eu1—N3	75.36 (10)	C41—C42—C43	119.8 (5)
O9—Eu1—O3	134.03 (9)	C41—C42—H42	120.1
O9—Eu1—O4	139.35 (10)	N3—C15—H15	118.1
O9—Eu1—N1	84.98 (10)	N3—C15—C14	123.9 (4)
O9—Eu1—N2	67.02 (8)	C14—C15—H15	118.1
O9—Eu1—C23	143.66 (11)	N3—C11—C10	117.0 (4)
O9—Eu1—O5	74.26 (9)	N3—C11—C12	120.9 (4)
O4—Eu1—N3	71.24 (10)	C12—C11—C10	122.1 (4)
O4—Eu1—O3	52.37 (8)	C38—C39—H39	119.9
O4—Eu1—N1	133.36 (9)	C40—C39—C38	120.2 (4)
O4—Eu1—N2	114.51 (9)	C40—C39—H39	119.9
O4—Eu1—C23	26.39 (9)	C42—C41—H41	120.0
O4—Eu1—O5	124.26 (9)	C40—C41—C42	120.0 (4)
O1—Eu1—N3	139.77 (10)	C40—C41—H41	120.0
O1—Eu1—O3	73.91 (9)	C25—C26—H26	120.3
O1—Eu1—O9	144.85 (10)	C27—C26—C25	119.4 (4)
O1—Eu1—O4	71.87 (10)	C27—C26—H26	120.3
O1—Eu1—N1	73.44 (10)	C39—C40—H40	119.8
O1—Eu1—N2	121.78 (9)	C41—C40—C39	120.4 (4)
O1—Eu1—C23	71.28 (10)	C41—C40—H40	119.8
O1—Eu1—O5	72.78 (9)	C3—C4—H4	120.1
N1—Eu1—N3	124.32 (10)	C3—C4—C5	119.8 (4)
N1—Eu1—N2	61.84 (10)	C5—C4—H4	120.1
N1—Eu1—C23	111.67 (11)	C14—C13—H13	120.8
N2—Eu1—C23	91.88 (10)	C12—C13—H13	120.8
O5—Eu1—N3	143.86 (9)	C12—C13—C14	118.4 (5)
O5—Eu1—O3	144.93 (9)	C1—C2—C3	117.9 (5)
O5—Eu1—N1	71.70 (10)	C1—C2—H2	121.0
O5—Eu1—N2	120.54 (9)	C3—C2—H2	121.0
O5—Eu1—C23	140.78 (10)	C6—C7—H7	120.6
O2^i^—Eu1—N3	79.81 (10)	C8—C7—C6	118.8 (4)
O2^i^—Eu1—O3	123.25 (9)	C8—C7—H7	120.6
O2^i^—Eu1—O9	79.32 (9)	N1—C5—C6	116.4 (4)
O2^i^—Eu1—O4	72.62 (9)	N1—C5—C4	121.6 (4)
O2^i^—Eu1—O1	103.81 (10)	C4—C5—C6	122.0 (4)
O2^i^—Eu1—N1	146.64 (10)	C15—C14—C13	118.2 (4)
O2^i^—Eu1—N2	134.08 (10)	C15—C14—H14	120.9
O2^i^—Eu1—C23	98.09 (11)	C13—C14—H14	120.9
O2^i^—Eu1—O5	75.75 (10)	C11—C12—H12	119.8
C15—N3—Eu1	119.7 (3)	C13—C12—C11	120.5 (4)
C15—N3—C11	118.1 (4)	C13—C12—H12	119.8
C11—N3—Eu1	121.6 (3)	C29—C28—H28	120.1
C23—O3—Eu1	92.5 (2)	C27—C28—C29	119.9 (4)
C37—O9—Eu1	141.9 (3)	C27—C28—H28	120.1
C23—O4—Eu1	93.6 (2)	C31—C32—H32	120.0
C16—O1—Eu1	139.7 (3)	C31—C32—C33	120.0 (5)
C1—N1—Eu1	119.5 (3)	C33—C32—H32	120.0
C1—N1—C5	117.1 (4)	C17—C18—H18	119.7
C5—N1—Eu1	122.9 (3)	C17—C18—C19	120.5 (5)
C6—N2—Eu1	120.8 (3)	C19—C18—H18	119.7
C10—N2—Eu1	120.3 (3)	C10—C9—H9	120.8
C10—N2—C6	118.6 (3)	C8—C9—C10	118.4 (4)
N2—C6—C7	121.8 (4)	C8—C9—H9	120.8
N2—C6—C5	116.7 (3)	C31—C36—H36	119.9
C7—C6—C5	121.5 (4)	C31—C36—C35	120.3 (5)
C24—C25—H25	119.7	C35—C36—H36	119.9
C26—C25—H25	119.7	C7—C8—C9	120.5 (4)
C26—C25—C24	120.6 (4)	C7—C8—H8	119.7
C18—C17—C16	120.5 (4)	C9—C8—H8	119.7
C18—C17—C22	119.3 (4)	C26—C27—H27	119.5
C22—C17—C16	120.2 (4)	C28—C27—C26	121.0 (4)
C25—C24—C23	120.6 (3)	C28—C27—H27	119.5
C25—C24—C29	119.4 (4)	C36—C35—H35	120.4
C29—C24—C23	120.0 (4)	C34—C35—C36	119.2 (6)
O3—C23—Eu1	61.47 (19)	C34—C35—H35	120.4
O3—C23—O4	121.5 (3)	C17—C22—H22	120.2
O3—C23—C24	120.0 (3)	C21—C22—C17	119.5 (5)
O4—C23—Eu1	60.06 (19)	C21—C22—H22	120.2
O4—C23—C24	118.4 (4)	O7—C30—C31	122.9 (4)
C24—C23—Eu1	175.2 (3)	O7—C30—O6	124.5 (4)
O1—C16—C17	116.5 (3)	O6—C30—C31	112.6 (4)
O2—C16—O1	124.8 (4)	C32—C33—H33	120.4
O2—C16—C17	118.7 (4)	C34—C33—C32	119.3 (5)
N2—C10—C11	117.7 (3)	C34—C33—H33	120.4
N2—C10—C9	121.9 (4)	C22—C21—H21	119.4
C9—C10—C11	120.4 (4)	C20—C21—C22	121.2 (5)
N1—C1—H1	117.7	C20—C21—H21	119.4
N1—C1—C2	124.7 (4)	C18—C19—H19	120.3
C2—C1—H1	117.7	C20—C19—C18	119.4 (5)
C32—C31—C36	120.0 (4)	C20—C19—H19	120.3
C32—C31—C30	122.1 (4)	C21—C20—C19	120.0 (5)
C36—C31—C30	117.8 (4)	C21—C20—H20	120.0
C43—C38—C37	119.6 (4)	C19—C20—H20	120.0
C43—C38—C39	118.8 (4)	C35—C34—H34	119.4
C39—C38—C37	121.5 (4)	C33—C34—C35	121.1 (5)
C38—C43—H43	119.6	C33—C34—H34	119.4
C38—C43—C42	120.8 (4)	C30—O6—H6	109.5
C42—C43—H43	119.6	Eu1—O5—H5A	110.7
O9—C37—C38	116.3 (4)	Eu1—O5—H5B	110.3
O8—C37—O9	123.1 (4)	H5A—O5—H5B	108.3
O8—C37—C38	120.6 (4)	C16—O2—Eu1^i^	168.9 (3)
C24—C29—H29	120.2		
			
Eu1—N3—C15—C14	170.4 (3)	C25—C24—C23—O3	161.1 (4)
Eu1—N3—C11—C10	10.9 (4)	C25—C24—C23—O4	−15.5 (6)
Eu1—N3—C11—C12	−169.6 (3)	C25—C24—C29—C28	−1.3 (6)
Eu1—O3—C23—O4	1.7 (4)	C25—C26—C27—C28	−0.6 (7)
Eu1—O3—C23—C24	−174.8 (3)	C17—C16—O2—Eu1^i^	138.9 (12)
Eu1—O9—C37—O8	14.9 (7)	C17—C18—C19—C20	−2.2 (8)
Eu1—O9—C37—C38	−163.1 (3)	C17—C22—C21—C20	0.1 (8)
Eu1—O4—C23—O3	−1.7 (4)	C24—C25—C26—C27	−1.0 (6)
Eu1—O4—C23—C24	174.8 (3)	C24—C29—C28—C27	−0.3 (7)
Eu1—O1—C16—C17	−178.2 (3)	C23—Eu1—N3—C15	−72.4 (3)
Eu1—O1—C16—O2	3.6 (7)	C23—Eu1—N3—C11	98.2 (3)
Eu1—N1—C1—C2	170.9 (3)	C23—Eu1—O9—C37	160.1 (4)
Eu1—N1—C5—C6	11.0 (4)	C23—Eu1—O1—C16	98.5 (4)
Eu1—N1—C5—C4	−170.2 (3)	C23—Eu1—N1—C1	98.4 (3)
Eu1—N2—C6—C7	173.7 (3)	C23—Eu1—N1—C5	−90.0 (3)
Eu1—N2—C6—C5	−6.5 (4)	C23—Eu1—N2—C6	121.9 (3)
Eu1—N2—C10—C11	5.1 (4)	C23—Eu1—N2—C10	−64.5 (3)
Eu1—N2—C10—C9	−173.7 (3)	C23—C24—C29—C28	176.9 (4)
N3—Eu1—O3—C23	79.5 (2)	C16—C17—C18—C19	−178.8 (4)
N3—Eu1—O9—C37	167.1 (4)	C16—C17—C22—C21	179.8 (4)
N3—Eu1—O4—C23	−79.4 (2)	C10—N2—C6—C7	0.1 (5)
N3—Eu1—O1—C16	95.0 (4)	C10—N2—C6—C5	179.8 (3)
N3—Eu1—N1—C1	176.6 (3)	C10—C11—C12—C13	178.2 (4)
N3—Eu1—N1—C5	−11.9 (3)	C10—C9—C8—C7	0.7 (6)
N3—Eu1—N2—C6	−173.5 (3)	C1—N1—C5—C6	−177.3 (3)
N3—Eu1—N2—C10	0.1 (2)	C1—N1—C5—C4	1.5 (6)
N3—Eu1—C23—O3	−90.9 (2)	C31—C32—C33—C34	0.5 (7)
N3—Eu1—C23—O4	90.8 (2)	C31—C36—C35—C34	−1.1 (7)
N3—C15—C14—C13	−0.1 (7)	C38—C43—C42—C41	0.3 (6)
N3—C11—C12—C13	−1.3 (6)	C38—C39—C40—C41	1.1 (7)
O3—Eu1—N3—C15	−100.0 (3)	C43—C38—C37—O9	7.5 (5)
O3—Eu1—N3—C11	70.6 (3)	C43—C38—C37—O8	−170.6 (4)
O3—Eu1—O9—C37	122.8 (4)	C43—C38—C39—C40	−0.5 (6)
O3—Eu1—O4—C23	0.9 (2)	C43—C42—C41—C40	0.3 (7)
O3—Eu1—O1—C16	125.6 (4)	C37—C38—C43—C42	176.3 (4)
O3—Eu1—N1—C1	110.6 (3)	C37—C38—C39—C40	−176.9 (4)
O3—Eu1—N1—C5	−77.8 (3)	C29—C24—C23—O3	−17.0 (6)
O3—Eu1—N2—C6	107.8 (3)	C29—C24—C23—O4	166.4 (4)
O3—Eu1—N2—C10	−78.6 (3)	C29—C28—C27—C26	1.2 (7)
O3—Eu1—C23—O4	−178.3 (4)	C3—C4—C5—N1	−0.5 (6)
O9—Eu1—N3—C15	112.1 (3)	C3—C4—C5—C6	178.2 (4)
O9—Eu1—N3—C11	−77.4 (3)	C42—C41—C40—C39	−1.0 (7)
O9—Eu1—O3—C23	125.0 (2)	C15—N3—C11—C10	−178.4 (3)
O9—Eu1—O4—C23	−115.8 (2)	C15—N3—C11—C12	1.1 (5)
O9—Eu1—O1—C16	−86.5 (4)	C11—N3—C15—C14	−0.5 (6)
O9—Eu1—N1—C1	−115.0 (3)	C11—C10—C9—C8	−179.2 (4)
O9—Eu1—N1—C5	56.6 (3)	C39—C38—C43—C42	−0.2 (6)
O9—Eu1—N2—C6	−88.6 (3)	C39—C38—C37—O9	−176.1 (4)
O9—Eu1—N2—C10	85.0 (3)	C39—C38—C37—O8	5.9 (6)
O9—Eu1—C23—O3	−83.6 (3)	C26—C25—C24—C23	−176.2 (4)
O9—Eu1—C23—O4	98.1 (3)	C26—C25—C24—C29	1.9 (6)
O4—Eu1—N3—C15	−44.4 (3)	C4—C3—C2—C1	1.3 (7)
O4—Eu1—N3—C11	126.2 (3)	C2—C3—C4—C5	−0.9 (7)
O4—Eu1—O3—C23	−0.9 (2)	C7—C6—C5—N1	177.2 (3)
O4—Eu1—O9—C37	−157.4 (4)	C7—C6—C5—C4	−1.6 (6)
O4—Eu1—O1—C16	70.6 (4)	C5—N1—C1—C2	−1.1 (6)
O4—Eu1—N1—C1	80.3 (3)	C5—C6—C7—C8	−179.4 (4)
O4—Eu1—N1—C5	−108.1 (3)	C14—C13—C12—C11	0.7 (7)
O4—Eu1—N2—C6	136.0 (2)	C12—C13—C14—C15	0.0 (6)
O4—Eu1—N2—C10	−50.5 (3)	C32—C31—C36—C35	1.1 (7)
O4—Eu1—C23—O3	178.3 (4)	C32—C31—C30—O7	167.6 (4)
O1—Eu1—N3—C15	−68.8 (3)	C32—C31—C30—O6	−14.4 (6)
O1—Eu1—N3—C11	101.7 (3)	C32—C33—C34—C35	−0.5 (8)
O1—Eu1—O3—C23	−80.2 (2)	C18—C17—C16—O1	−5.0 (6)
O1—Eu1—O9—C37	−11.9 (5)	C18—C17—C16—O2	173.4 (4)
O1—Eu1—O4—C23	84.3 (2)	C18—C17—C22—C21	−0.5 (7)
O1—Eu1—N1—C1	37.0 (3)	C18—C19—C20—C21	1.7 (9)
O1—Eu1—N1—C5	−151.5 (3)	C9—C10—C11—N3	168.4 (3)
O1—Eu1—N2—C6	52.9 (3)	C9—C10—C11—C12	−11.1 (6)
O1—Eu1—N2—C10	−133.6 (3)	C36—C31—C32—C33	−0.7 (6)
O1—Eu1—C23—O3	91.6 (2)	C36—C31—C30—O7	−9.4 (6)
O1—Eu1—C23—O4	−86.8 (2)	C36—C31—C30—O6	168.5 (4)
O1—C16—O2—Eu1^i^	−42.9 (16)	C36—C35—C34—C33	0.8 (8)
N1—Eu1—N3—C15	−174.6 (3)	C22—C17—C16—O1	174.7 (4)
N1—Eu1—N3—C11	−4.1 (3)	C22—C17—C16—O2	−7.0 (6)
N1—Eu1—O3—C23	−153.3 (2)	C22—C17—C18—C19	1.5 (7)
N1—Eu1—O9—C37	39.7 (4)	C22—C21—C20—C19	−0.7 (9)
N1—Eu1—O4—C23	40.5 (3)	C30—C31—C32—C33	−177.7 (4)
N1—Eu1—O1—C16	−141.0 (4)	C30—C31—C36—C35	178.2 (4)
N1—Eu1—N2—C6	8.3 (2)	O5—Eu1—N3—C15	78.5 (3)
N1—Eu1—N2—C10	−178.2 (3)	O5—Eu1—N3—C11	−111.0 (3)
N1—Eu1—C23—O3	28.9 (3)	O5—Eu1—O3—C23	−98.9 (3)
N1—Eu1—C23—O4	−149.5 (2)	O5—Eu1—O9—C37	−32.7 (4)
N1—C1—C2—C3	−0.3 (7)	O5—Eu1—O4—C23	137.4 (2)
N2—Eu1—N3—C15	−176.5 (3)	O5—Eu1—O1—C16	−65.5 (4)
N2—Eu1—N3—C11	−6.0 (2)	O5—Eu1—N1—C1	−39.9 (3)
N2—Eu1—O3—C23	146.2 (3)	O5—Eu1—N1—C5	131.7 (3)
N2—Eu1—O9—C37	101.2 (4)	O5—Eu1—N2—C6	−34.9 (3)
N2—Eu1—O4—C23	−33.1 (3)	O5—Eu1—N2—C10	138.6 (2)
N2—Eu1—O1—C16	178.7 (4)	O5—Eu1—C23—O3	116.1 (2)
N2—Eu1—N1—C1	178.5 (3)	O5—Eu1—C23—O4	−62.2 (3)
N2—Eu1—N1—C5	−10.0 (3)	O2^i^—Eu1—N3—C15	30.6 (3)
N2—Eu1—C23—O3	−31.5 (2)	O2^i^—Eu1—N3—C11	−158.9 (3)
N2—Eu1—C23—O4	150.2 (2)	O2^i^—Eu1—O3—C23	16.0 (3)
N2—C6—C7—C8	0.3 (6)	O2^i^—Eu1—O9—C37	−110.7 (4)
N2—C6—C5—N1	−2.6 (5)	O2^i^—Eu1—O4—C23	−164.3 (2)
N2—C6—C5—C4	178.6 (3)	O2^i^—Eu1—O1—C16	4.5 (4)
N2—C10—C11—N3	−10.4 (5)	O2^i^—Eu1—N1—C1	−53.1 (4)
N2—C10—C11—C12	170.0 (3)	O2^i^—Eu1—N1—C5	118.5 (3)
N2—C10—C9—C8	−0.3 (6)	O2^i^—Eu1—N2—C6	−134.9 (2)
C6—N2—C10—C11	178.8 (3)	O2^i^—Eu1—N2—C10	38.6 (3)
C6—N2—C10—C9	0.0 (5)	O2^i^—Eu1—C23—O3	−166.5 (2)
C6—C7—C8—C9	−0.7 (6)	O2^i^—Eu1—C23—O4	15.1 (2)

Symmetry code: (i) −*x*+1, −*y*+2, −*z*+1.

### Hydrogen-bond geometry (Å, º)

**Table:**

*D*—H···*A*	*D*—H	H···*A*	*D*···*A*	*D*—H···*A*
O6—H6···O8^ii^	0.82	1.76	2.582 (4)	178
O5—H5*A*···O8	0.87	1.98	2.796 (4)	155
O5—H5*B*···O4^i^	0.87	1.95	2.768 (4)	155

Symmetry codes: (i) −*x*+1, −*y*+2, −*z*+1; (ii) *x*, *y*−1, *z*−1.
