# Supplementary figures and images for: Crystal structure of di-μ-benzato-κ4 O:O′-bis­[aqua­(benzato-κO)(benzato-κ2 O,O′)(2,2′:6′,2′′-terpyridine-κ3 N,N′,N′′)europium(III)]–benzoic acid (1/2)
Source: Acta Crystallogr Sect E Struct Rep Online. 2014 Aug 13;70(Pt 9):m328–9. doi: 10.1107/S1600536814018182 (PMC4186121; doi:10.1107/S1600536814018182)

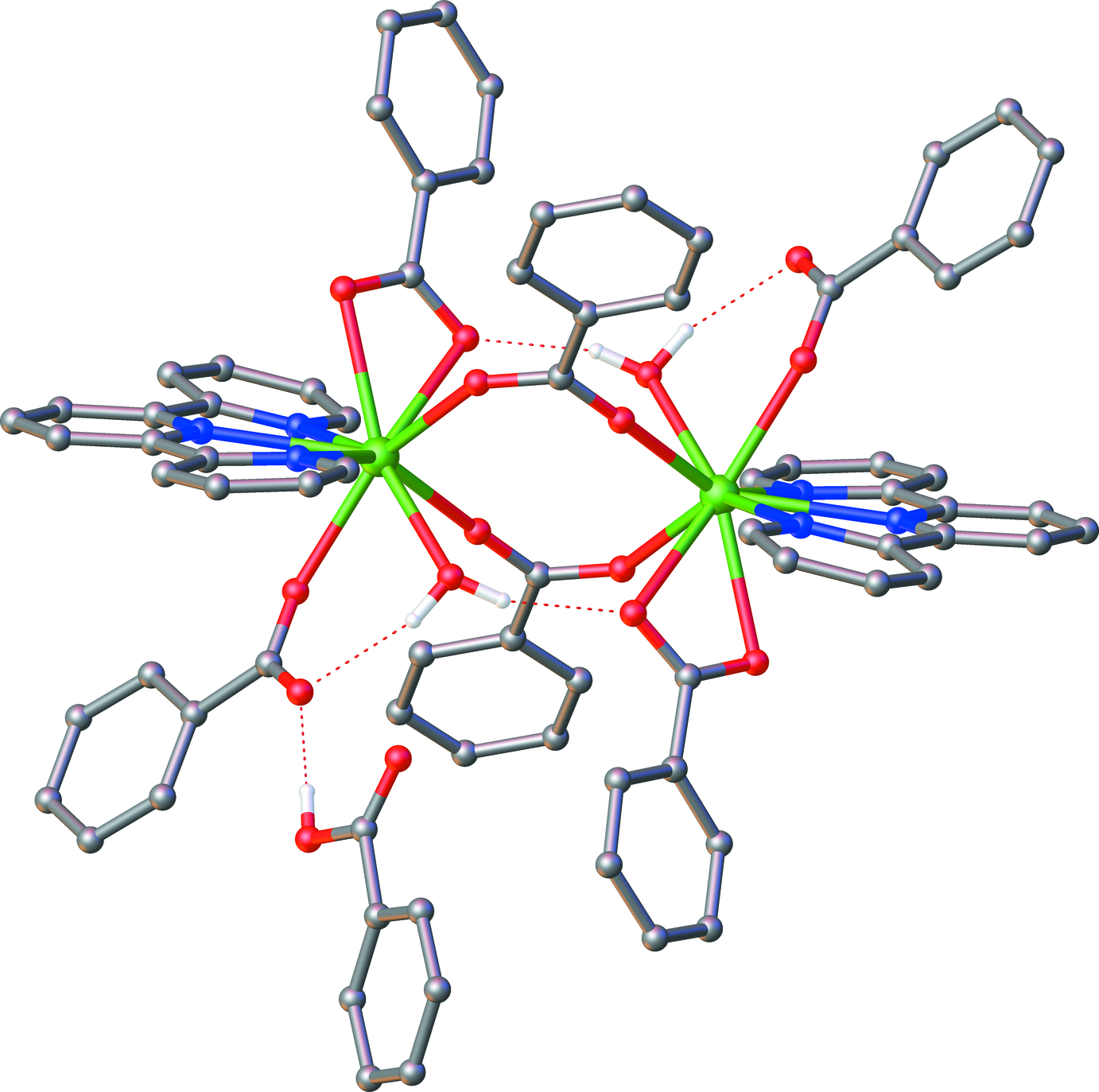

Supplement: Supplementary file 3 [file e-70-0m328-fig1.tif]
